# The accuracy of an electronic nose to diagnose tuberculosis in patients referred to an expert centre

**DOI:** 10.1371/journal.pone.0276045

**Published:** 2023-02-07

**Authors:** Rosarito Coronel Teixeira, Luis Gómez, Eva González, Nilda Jiménez de Romero, Felipe González, Sarita Aguirre, Martin Boeree, Robin Janssen, Cecile Magis-Escurra

**Affiliations:** 1 National Institute of Respiratory Diseases and the Environment (INERAM), Asunción, Paraguay; 2 Department of Respiratory Diseases, Radboud University Medical Centre -TB Expert Centre Dekkerswald, Nijmegen—Groesbeek, The Netherlands; 3 National Reference Laboratory of Public Health Laboratory (LCSP), Asunción, Paraguay; 4 National Tuberculosis Control Program (NTCP), Asunción, Paraguay; University of Pisa, ITALY

## Abstract

**Introduction:**

An electronic nose (eNose) device has shown a high specificity and sensitivity to diagnose or rule out tuberculosis (TB) in the past. The aim of this study was to evaluate its performance in patients referred to INERAM.

**Methods:**

Patients aged ≥15 years were included. A history, physical examination, chest radiography (CRX) and microbiological evaluation of a sputum sample were performed in all participants, as well as a 5-minute breath test with the eNose. TB diagnosis was preferably established by the gold standard and compared to the eNose predictions. Univariate and multivariate logistic regression analyses were performed to assess potential risk factors for erroneous classification results by the eNose.

**Results:**

107 participants with signs and symptoms of TB were enrolled of which 91 (85.0%) were diagnosed with TB. The blind eNose predictions resulted in an accuracy of 50%; a sensitivity of 52.3% (CI 95%: 39.6–64.7%) and a specificity of 36.4% (CI 95%: 12.4–68.4%). Risk factors for erroneous classifications by the eNose were older age (multivariate analysis: OR 1.55, 95% CI 1.10–2.18, *p* = 0.012) and antibiotic use (multivariate analysis: OR 3.19, 95% CI 1.06–9.66, *p* = 0.040).

**Conclusion:**

In this study, the accuracy of the eNose to diagnose TB in a tertiary referral hospital was only 50%. The use of antibiotics and older age represent important factors negatively influencing the diagnostic accuracy of the eNose. Therefore, its use should probably be restricted to screening in high-risk communities in less complex healthcare settings.

## Introduction

Although tuberculosis (TB) may seem a ‘silent pandemic’ compared to COVID-19, it is responsible, yearly, for 10 million cases and 1.2 million notified deaths [1, 2]. TB elimination is complex. On the one side due to different elements in the ‘cascade of care’ to establish the diagnosis but also because of factors concerning the bacteria itself as slow duplication and drug resistance which complicates treatment [1].

The “holy grail” to diagnose TB is a portable and rapid ‘point-of-care’ test providing a result within minutes [3]. Such a rapid test will never replace the current gold standard (positive culture of *Mycobacterium tuberculosis* complex) but may be used as a screening tool to either rule out TB or detect individuals with active disease. All conventional diagnostic methods, such as chest radiography (CXR), or sputum based test as Ziehl Neelsen (ZN) staining, GeneXpert MTB/RIF and culture of MTB are either expensive, time consuming, not widely available, and labour intensive [1].

An electronic nose (eNose) is a small, portable and a potentially rapid diagnostic ‘point-of-care’ device, that can detect gases in human breath and compares vectors derived from volatile organic compound (VOCs) from exhaled breath samples to a machine learning algorithm to diagnose a patient with TB [4]. Analysers for exhaled breath can be separated in two groups: 1. Systems for detecting and measuring a set of specific VOC’s. Usually, these systems comprise of a mobile unit for collecting the gas sample and another (fixed) unit for analysing it and separating the individual components. Separation techniques typically include GC-MS or IMS. 2. Electronic noses, often portable, possessing multiple different a-specific sensors (e.g. metal-oxide or conducting polymer) for measuring an integrated breath profile. In this case, Machine Learning techniques are applied for separating breath profiles of sick and healthy individuals. In order to accomplish this, a classifier (e.g. Artificial Neural Network, Random Forest, Support Vector Machine) has to be trained accordingly [5]. Until now, several studies have been performed with the eNose, which showed promising results [6–9]. In Paraguay, the eNose has been tested in adults (>18 years) with pulmonary TB, asthma or COPD and healthy controls, showing a high sensitivity (91%) and specificity (93%) to diagnose TB [10]. The eNose was also tested in an indigenous population, demonstrating its usefulness as a rule-out TB test in a remote area [11]. Additional studies testing the eNose in different settings are needed.

The aim of this study was to assess the accuracy of the eNose to classify individuals with signs and symptoms compatible with TB (in- and outpatient clinic) referred to the Paraguayan National Reference Center for respiratory diseases and TB (INERAM).

## Methods

### Study design and setting

A prospective study was performed in hospitalized and ambulatory patients from January 2016 to December 2017.

### Participants

Participants, aged ≥ 15 years, presenting with respiratory symptoms for more than 15 days or having started anti-TB treatment already (<3 days before inclusion) were included. Authorization by parents and/or guardians was provided for participants under 18 years old. In Human Immunodeficiency Virus (HIV) positive individuals, the duration of respiratory symptoms was not considered. Exclusion criteria were respiratory failure or not willing or unable to sign informed consent.

### Data collection

Patient demographics and clinical data (age, gender, BMI), information on co-morbidities (HIV, diabetes mellitus, asthma, chronic obstructive lung disease (COPD), or arterial hypertension), smoking and alcohol habits, drug abuse, co-medication (antibiotic use and/or TB treatment), food and beverage intake, and dental status (good condition: complete parts, no cavities or seals; regular: complete parts, presence of seals; bad: incomplete parts, presence of caries, incomplete seals) were recorded. Physical examination, a CXR, microbiological examination of sputum or biopsies and breath sampling with the eNose were performed in all participants. Microbiological examinations and breath tests conducted on the same day. TB symptoms (cough, fever, dyspnoea, night sweat, lymphadenopathy and/or haemoptysis) were noted. The study physicians assessed the CXRs for cavities, infiltrates, atelectasis and pleural effusion. All participants provided at least one sputum sample (produced spontaneously or induced by nebulization of 4 mL of hypertonic saline). ZN staining and mycobacterial culture (solid Ogawa-Kudoh medium) were performed in the laboratory of INERAM. In some cases, GeneXpert was performed according to the national guideline. The GeneXpert MTB/RIF® (Cepheid) samples were processed by the National Reference Laboratory (LCSP). The sputum samples were also evaluated for mycosis and common pathogens. Pulmonary TB (PTB) diagnosis was established by gold standard (positive culture of MTB complex). In case of a negative culture result, diagnosis was set by other strong supporting evidence (e.g. ZN positive, and/or MTB detected by GeneXpert [12]. In case no bacteriological prove was found a diagnosis was called a ‘clinical diagnosis’. In this last category, the improvement of clinical symptoms with anti-TB treatment was evaluated after two months (the initial phase of treatment). Extra-pulmonary TB (EPTB) patients were diagnosed by microbiological confirmation (positive culture of MTB) or with histopathological or chemical evidence supporting TB (in biopsies, pus or pleural fluid), or showing improvement of clinical condition after two months of anti-TB treatment. All participants were followed at the outpatient clinic. TB patients received anti-TB treatment according to the national guideline [13].

### Breath sampling with the eNose

All participants underwent five-minute breath sampling using a nose clamp, through the eNose (Aeonose^TM^ device, Zutphen, The Netherlands). The breath test measurements were done in the morning, always in the same place, in a room free of odours of gasses and alcohol and without dust. Cleaning of the eNose device on the outside was not performed unless it was needed because of stains on the device from a previous user. After every breath sampling a clean burn (approximately 10 minutes) was performed inside the device by heating of the metal sensors to 280 degrees Celsius, as part of the complete cycle process. Without this clean burn the eNose device cannot be used for another participant. Time of last meal/beverage in take/ medication or cigarette were recorded. All participants used a nose clamp and provided a breath sample by in- and exhaling for 5 min, through the Aeonose^TM^. The same device was used throughout the whole study. During sampling process, 36 measurement cycles, each containing 64 data points, were recorded per sensor. By performing this, each patient’s measurement comprised a data matrix with thousands of records. Proper reproducibility of the results is possible due to the sensor’s temperature control. However, even for sensors produced on the same wafer, thickness and ageing differences can cause small variations between sensors and AeonoseTM devices over time. This phenomenon is cope by normalizing the data. The data is then compressed using a Tucker 3-like algorithm. This results into a vector of 10 components per patient, redundant information and noise are remove. These resulting vectors and the results of the classification are used to train an artificial neural network (ANN). A double cross-validation was applied using the Leave-10%-Out method to minimize the risk of systematic errors [10].

The analysis of the breath samples was done by *The eNose Company*. The company did not take part in study design and logistics. To establish a new ‘training data set’ to train and optimize the previously used neural network (NN) from our calibration study [10], 31 de-blinded participants from this new cohort were added to the former training data set. For full description of the training and internal validation of the NN; see previous published research [6].

The data of the remaining study participants (n = 76) was kept blind to The eNose Company. These participants of the ‘blind data set’ were analysed in the optimized NN and then classified by the eNose Company as “TB yes” or “TB no”.

### Statistical analysis

Statistical analysis was performed by using SPSS version 25.0 software (IBM Corporation, Armonk, NY, USA). Continuous variables were expressed as the mean (with standard deviation) or median (with range), and categorical variables were expressed as frequency (with percentage). Univariate testing of continuous variables was performed using Student t-test in case of normal distribution, and non-parametric tests for non-normal distribution. Categorical variables were analysed using X^2^ testing or Fisher’s exact test. Binary logistic regression analysis was performed to identify possible risk factors for wrong predictions in the NN algorithm. Variables with a p value ≤0.10 in univariate analysis were selected for multivariate logistic regression analysis. To prevent overfitting, the number of variables in the multivariate analysis was restricted by the number of patients with the outcome. All tests were two-sided, and a *p* value of ≤ 0.05 was considered statistically significant.

### Inclusivity in global research

Additional information regarding the ethical, cultural, and scientific considerations specific to inclusivity in global research is included in the S1 Questionnaire.

## Results

During the study period, 107 participants were enrolled with a mean age of 37 years and 70% being male (Table 1). TB was diagnosed in 91/107 (85%) of the participants. The most common TB characteristic was cough (87.9%), followed by fever (77.6%), dyspnoea (73.8%) and night sweat (72.0%). Four HIV positive TB patients were included and were on antiretroviral therapy. Fifty-six participants (52.3%) started TB treatment for ≤3 days when included in this study and providing the breath sample. Two participants received TB treatment in the past (>3 years ago). Table 2 shows microbiological outcomes of all participants. TB diagnosis was established by gold standard in 67 cases (74%). Twenty-six percent (24/91) were diagnosed differently; five PTB with ZN positive sputum only and another five were ‘clinical diagnosis’. Fourteen patients with EPTB (pleural or lymph node) also lacked bacteriological confirmation. The remaining group of 16/107 (15.0%) participants had an alternative diagnosis (e.g. pneumonia or chronic obstructive pulmonary disease (COPD) exacerbation).

**Table 1:** Participant demographic table.

	Total group of participants (n = 107)
Mean age, years (range)	37 (15–83)
Sex, male (%)	75 (70.1%)
Body Mass Index (range)	21 (14–32)
Habits	
Current smoker	33 (30.8%)
Alcohol (3 times per week)	3 (2.8%)
Drug abuse^+^	10 (9.3%)
Dental status	
Good	14 (13.1%)
Regular	61 (57.0%)
Bad	32 (29.9%)
Median time of food–beverage intake before test, hours (range)	3 (0.1–20)
Co-morbidity	
HIV	4 (3.7%)
Diabetes Mellitus	13 (12.1%)
Asthma / COPD	2 (1.9%) / 20 (18.7%)
Arterial hypertension	5 (4.7%)
Antibiotic use*, yes	68 (63.5%)
TB treatment, yes	56 (52.3%)
Symptoms	
Cough	94 (87.9%)
Fever	83 (77.6%)
Dyspnoea	79 (73.8%)
Night sweat	77 (72.0%)
Lymphadenopathy	7 (6.5%)
Haemoptysis	7 (6.5%)
Chest X-ray results	
Cavities	56 (52.3%)
Infiltrate	85 (79.4%)
Atelectasis	15 (14.0%)
Pleural effusion	37 (34.6%)

* General antibiotics (penicillin, quinolones).

^+^ Drug use: Marihuana, crack, glue sniffing cobbler’s tail.

**Table 2:** Microbiological results.

	Total group of participants (n = 107)
**Mycobacterial culture**	
Positive	67 (62.6%)
Negative	31 (29.0%)
Not done*	6 (5.6%)
Missing	3 (2.8%)
**ZN staining**	
Positive	56 (52.3%)
Negative	39 (36.4%)
Not done*	6 (5.6%)
Missing	6 (5.6%)
**GeneXpert**	
Positive	19 (17.8%)
Negative	11 (10.3%)
Not done	77 (72.0%)
**Histopathology**	
Positive	11 (10.3%)
Negative	1 (0.9%)
**Common pathogens/mycosis**	
Normal flora	95 (89.0%)
*Acinetobacter baumannii*	2 (1.9%)
*Klebsiella pneumoniae*	1 (0.9%)
*Enterobacter spp*	1 (0.9%)
*Serratia marcescens*	1 (0.9%)
*Paracocidioidomycosis*	1 (0.9%)
*Pseudomonas*	3 (2.8%)
*Staphylococcus spp*	1 (0.9%)
Presence of hyphae	2 (1.9%)

*6 patients were unable to produce sputum.

**Fig 1:**
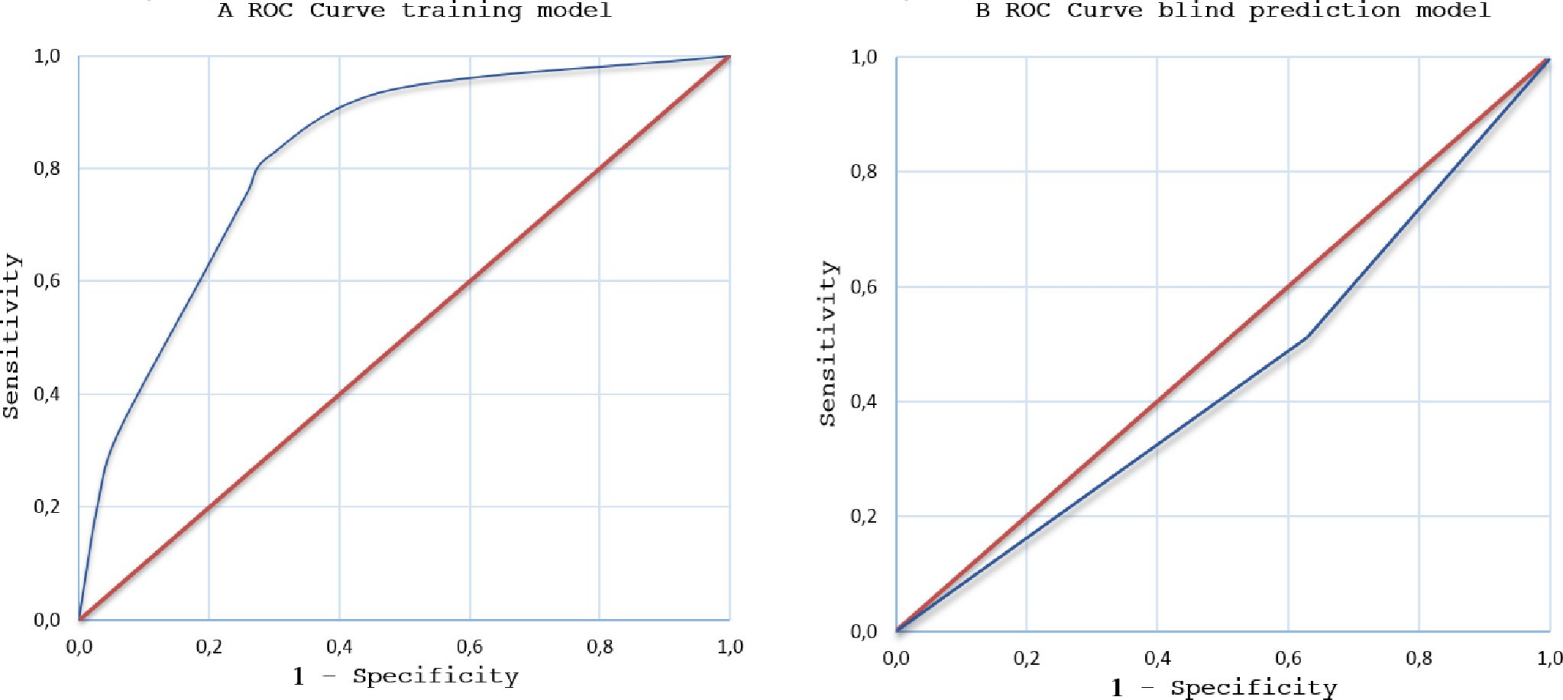
ROC curve of the training and the blind-prediction model.

**Fig 2:**
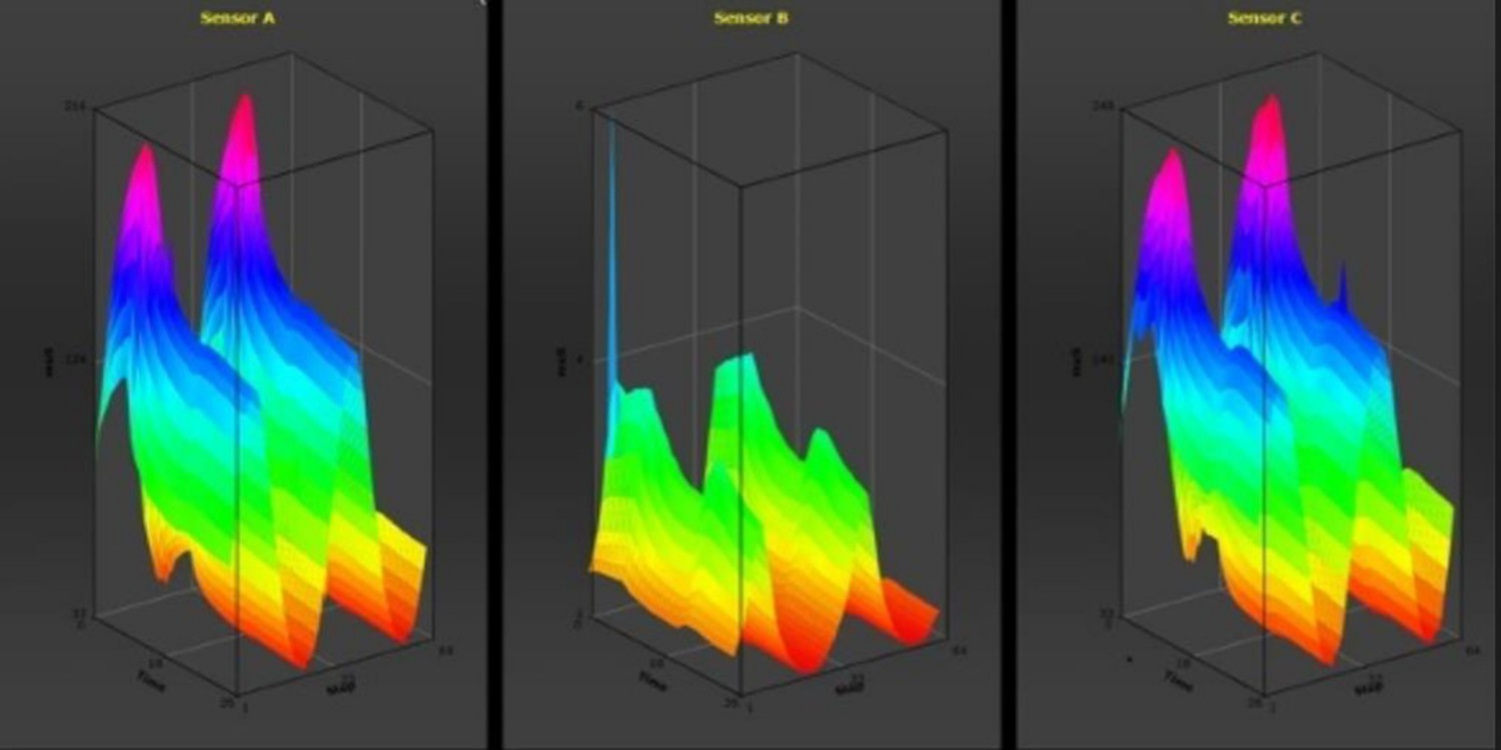
Raw sensor signals from the electronic nose.

The sensitivity and specificity of the NN in the ‘training data set’ (n = 137) were 80% (Confidence Interval (CI) 95%: 68.3–89.0%) and 84% (CI 95%: 74.0–91.0%), respectively. The positive predicted value (PPV) was 80% (CI 95%: 68.3–89.0%) and the negative predictive value (NPV) was 84% (CI 95%:74.0–89.0%). Fig 1A shows the ROC curve for this ‘training data set’. Twenty-four wrongly classified participants (24/137 (17.5%); 13/24 (54.2%) were receiving antibiotics and 5/24 (20.8%) were obese (Body Mass Index (BMI) ≥ 25). Fig 2 shows a raw sensor signals from the electronic nose.

The analysis of the NN with the ‘blind data set’ (n = 76 participants) resulted in 38 correct and 38 wrong classifications, yielding a sensitivity and specificity of 52.3% (CI 95%: 39.6–64.7%) and 36.4% (CI 95%: 12.4–68.4%) and an accuracy of 50%. The Positive Predicted Value (PPV) was 83.0% (CI 95%: 67.4–92.3%) with a Negative Predictive Value (NPV) of 11.4% (CI 95%: 3.7–27.7%). Fig 1B shows the ROC curve of the blind-prediction. Of the false positive cases (n = 7), three had comorbidities and six were under antibiotic treatment (other than anti-TB treatment) for at least 2 days. All false positive participants were admitted to the in-patient clinic and later discharged with a final diagnosis of pneumonia. Of the 31 false negative cases, 11/31 (35%) had comorbidities, 25/31 (81%) were under antibiotic treatment and 23/31 (74%) were under anti-TB treatment for at least 48 hours. Older age (35 years vs. 44 years, p = 0.028) and the use of antibiotics (60.5% vs. 81.8%, p = 0.043) were represented significantly different between the correct and wrongly classified participants (Table 3).

**Table 3:** Participant characteristics of the correct prediction group versus the wrong prediction group.

	Total participants of the blind prediction group (n = 76)	Correct prediction (n = 38)	Wrong prediction (n = 38)	*p* value
Median age, years (range)	38 (15–83)	35 (15–62)	44 (15–83)	0.028
Sex, male (%)	51 (67.1%)	24 (63.2%)	27 (71.1%)	0.464
BMI (SD)	21 (14–31)	21 (14–31)	21 (17–29)	0.966
Habits				
Current smoker	25 (32.8%)	16 (42.1%)	11 (28.9%)	0.231
Alcohol use*	14 (18.4%)	8 (21.1%)	6 (15.8%)	0.554
Drug use^+^	8 (10.5%)	6 (17.6%)	2 (5.3%)	0.138
Dental status				0.322
Good	7 (9.2%)	5 (13.2%)	2 (5.3%)	
Regular	46 (60.5%)	24 (63.2%)	22(57.9%)	
Bad	23 (30.2%)	9 (23.7%)	14 (36.8%)	
Median time of food–beverage intake before test, hours (range)	2 (0–13)	2 (0–13)	2 (0–12)	0.867
Co-morbidity, yes	27 (35.5%)	13 (34.2%)	14 (36.8%)	0.811
Antibiotic^‡^ use, yes	54 (71.1%)	23 (60.5%)	31 (81.8%)	0.043
TB drugs use, yes	40 (52.6%)	17 (44.7%)	23 (60.5%)	0.168
Symptoms^§^				
Cough	69 (90.8%)	33 (86.8%)	36 (94.7%)	0.430
Fever	65 (85.5%)	33 (86.8%)	32 (84.2%)	0.744
Dyspnoea	63 (82.9%)	32 (84.2%)	31 (81.6%)	0.761
Night sweat	62 (81.6%)	30 (78.9%)	32 (84.2%)	0.554
Chest X-ray results				
Cavities	42 (55.3%)	20 (54.1%)	22 (57.9%)	0.738
Infiltrate	60 (78.9%)	29 (78.4%)	31 (81.6%)	0.729
Atelectasis	12 (15.8%)	5 (13.5%)	7 (18.4%)	0.562
Pleural effusion	25 (32.9%)	11 (29.7%)	14 (36.8%)	0.514
Breath sampling				
With difficulty^ǁ^	8 (10.5%)	5 (13.2%)	3 (7.9%)	0.711

* Alcohol use

+ Drug use: Marihuana, crack, glue sniffing cobbler’s tail.

‡ General antibiotics (penicillin, quinolones).

§ Symptoms: Patients could have multiple symptoms.

ǁ Breath sampling with difficulty.

Table 4 shows the univariate and multivariate analysis of the possible risk factors associated with wrong predictions of TB diagnosis. Older age (univariate: OR 1.50, 95% CI 1.08–2.06, *p* = 0.016; multivariate: OR 1.55, 95% CI 1.10–2.18, *p* = 0.012) and the use of antibiotics (other than anti-TB treatment) for more than 24 hours (univariate: OR 2.89, 95% CI 1.01–8.23, p = 0.047; multivariate: OR 3.19, 95% CI 1.06–9.66, *p* = 0.040) were identified as risk factors associated with significant higher risk of erroneous classifications with the eNose.

**Table 4:** Univariate and multivariate logistic regression analysis for incorrect classifications in the neural network algorithm (blind prediction data set (n = 76)).

	Univariate		Multivariate	
	OR (95% CI)	*p* value	OR (95% CI)	*p* value
Gender				
Male	Ref			
Female	0.70 (0.27–1.83)	0.465		
Age (per 10 years increase)	1.50 (1.08–2.06)	0.016	1.55 (1.10–2.18)	0.012
Body mass index (kg/m^2^)	1.00 (0.87–1.14)	0.965		
Tobacco use				
No	Ref			
Yes	0.56 (0.22–1.45)	0.233		
Time last meal				
≥5 hours	Ref			
0–4 hours	5.46 (0.61–49.15)	0.130		
Dental status				
Good	Ref			
Bad	3.89 (0.62–24.52)	0.148		
Regular	2.29 (0.40–13.04)	0.350		
Comorbidities				
No	Ref			
Yes	1.12 (0.44–2.87)	0.811		
General antibiotic use				0.040
No	Ref		Ref	
yes	2.89 (1.01–8.23)	0.047	3.19 (1.06–9.66)	
TB Medication				
No	Ref	0.170		
Yes	1.89 (0.76–4.72)			
Germs				
Normal mouth flora	Ref			
Bacteria	0.18 (0.02–1.65)	0.130		
Mycosis	1.83 (0.16–21.15)	0.629		
TB				
No	Ref			
Yes, gold standard	0.57 (0.15–2.20)	0.416		
Yes, other*	0.38 (0.08–1.90)	0.239		

OR odds ratio; CI confidence interval.

*TB yes other: diagnosis not based on gold standard.

## Discussion

We evaluated the performance of the eNose in a referral hospital to diagnose TB in ill patients presenting with respiratory symptoms. We showed that in this study the accuracy of the eNose device was disappointingly only 50%. In this setting the eNose did not perform well enough according to the requisites for new screening tools or tests [3]. Older age and the use of antibiotics are significant risk factors for an incorrect prediction by the eNose with a very high OR for antibiotics use before TB diagnosis.

VOCs are unique to an individuals’ metabolism and may provide valuable information about the health condition of a subject. However, both host factors (genetics, co-morbidity, type of pathogen) and external factors (medication, toxic habits, food intake) may alter their breath prints, which could possibly result both in FN or FP classifications of the NN [14–16]. The use of antibiotics in a person with signs and symptoms compatible with TB is known to negatively influence the accuracy of conventional methods to diagnose TB, resulting in diagnostic delays in daily practice [17–22]. In our study, the use of antibiotics also significantly influenced the accuracy of the eNose device in a negative way. Even though there are several studies showing that co-medication influences breath prints, to the best of our knowledge, there are no publications specifically indicating the influence of antibiotics to an individuals’ VOCs.

We found that older age of the patients was also a risk factor for incorrect prediction by the eNose. Several studies have shown an association between age and breath prints [23–26]. The precise impact of ageing on metabolism is a topic that is widely discussed in the literature. It is not unreasonable to expect that this factor influences the concentrations of some metabolites and thereby altering breath prints [16].

In contrast to antibiotic use, anti-TB medication (< 3 days before inclusion of the participant) did not have a statistically significant impact on the predictive value of the eNose in this study. This may be explained because active TB is a disease that develops slowly over the course of weeks to months and requires a long duration of treatment to sterilize the patient from the bacteria. For that reason, it is understandable that a patients’ metabolism is not changing rapidly within the course of a few days. Therefore, the recent start of anti-TB treatment did not negatively influence the eNose predictions in our study cohort. We also did not find any association between BMI and incorrect predictions, even though there is evidence available that patients with a high BMI have different breath prints and a higher risk of false-positive test results compared to patients with a normal or low BMI [10, 23, 27, 28]. Dental status, time to last meal/ drinks, smoking and drug use neither appeared to be risk factors for a wrong prediction by the eNose in this study.

Our study has some limitations. Firstly, the sample size was small and consisted only of a small group of patients with an alternative diagnosis. Therefore, we might have introduced a design bias as the new NN ‘training data set’ might not have had enough pneumonia patients to train the new diagnostic algorithm correctly as the previous cohort consisted of PTB patients, patients with obstructive airway disease and healthy controls. Analysing larger cohorts will increase the accuracy of the eNose by establishing a more robust neural network algorithm. Secondly, the gold standard to establish TB diagnosis was not accomplished in all patients (mainly patients with EPTB) and it is possible that these patients in fact do not have active TB and the eNose classification was correctly made.

## Conclusion

In this study, the accuracy of the eNose to diagnose TB in a tertiary referral hospital was only 50%. Factors associated with wrong predictions by the eNose were antibiotic use and older age of the participants. For complex referral patients who mostly have used antibiotics to rule out a simple pneumonia first, as recommended by the WHO, the eNose showed not to be useful. The eNose is likely to be more suitable as a TB rule-out test in less complex healthcare settings.

## Supporting information

S1 Questionnaire (DOCX)

S1 Database (XLSX)
